# Antibacterial Activities and Underlying Mechanisms of the Compound SYAUP-491 against *Xanthomonas oryzae* pv. *oryzae*

**DOI:** 10.3390/molecules29061413

**Published:** 2024-03-21

**Authors:** Lina Li, Yuxin Wang, He Liu, Wei Liu, Xinchen Zhang, Mengnan An, Miao Yu, Yuanhua Wu, Xinghai Li, Jianzhong Wang

**Affiliations:** 1 College of Plant Protection, Shenyang Agricultural University, No. 120 Dongling Road, Shenhe District, Shenyang 110866, China; lln231118@163.com (L.L.); 18624512481@163.com (Y.W.); 2023500024@syau.edu.cn (H.L.); liuwei871016@163.com (W.L.); zhangxinchen999@163.com (X.Z.); anmengnan@syau.edu.cn (M.A.); ym13998369909@163.com (M.Y.); wuyh09@syau.edu.cn (Y.W.); 2 Institute of Agricultural Quality Standards and Testing Technology, Liaoning Academy of Agricultural Sciences, No. 84 Dongling Road, Shenhe District, Shenyang 110866, China

**Keywords:** antibacterial activity, *Xanthomonas oryzae* pv. *oryzae*, mechanism, alkyl sulfonamide

## Abstract

SYAUP-491 is a novel alkyl sulfonamide. In this study, in vivo and in vitro tests were performed along with a proteomic analysis to determine the effects and underlying mechanisms of the antibacterial activity of SYAUP-491 against the causative agent of bacterial leaf blight in rice. The antibacterial test results suggested that SYAUP-491 exhibited significant activities against *Xanthomonas oryzae* pv. *oryzae* (Xoo) in vitro and in vivo. The minimal EC_50_ values reached 6.96 μg/mL and the curative activity reached 74.1%. Detailed studies demonstrated that SYAUP-491 altered membrane permeability and caused morphological changes. Based on proteomics results, SYAUP-491 might inhibit bacterial protein synthesis. SYAUP-491 may disrupt and alter cell membrane permeability and could further act on ribosomes in the bacterial body. Given the above results, SYAUP-491 could serve as a new lead compound in the research of antibacterial control of plant pathogenic bacterial disease.

## 1. Introduction

Rice is one of the most important food crops in the world, consumed by over half of the world’s population [1]. A major threat to this staple crop is bacterial leaf blight, which is caused by *Xanthomonas oryzae* pv. *oryzae* (Xoo). This threat arises because bacterial leaf blight occurs frequently, results in large losses, and is hard to suppress [2,3,4,5]. Specifically, bacterial leaf blight cannot be adequately controlled using current commercial pesticides such as zinc thiazole, thiodiazole copper, and zhongshengmycin. Moreover, the long-term application of a single pesticide in large quantities can lead to resistance and associated environmental problems. Therefore, there is an urgent need to develop better ways to control bacterial plant diseases, such as the use of antibacterial compounds with novel structures.

In recent decades, sulfonamides have attracted widespread attention in the field of pharmacology due to their anticancer [6,7], antimicrobial [8,9], anti-HIV [10], anti-thyroid [11], anti-diabetic [12,13], and diuretic properties [14,15]. Moreover, sulfonamides are often used in mass application veterinary treatments due to their low cost and broad-spectrum activity (antibacterial and anticoccidial) [16]. In additin, sulfonamides are used in agriculture as fungicides [17,18], herbicides [19], and herbicide safeners [20].

**Figure 1:**
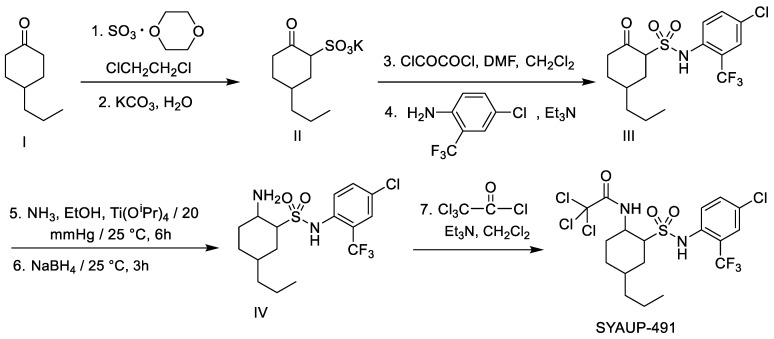
Synthetic route of the compound SYAUP-491.

Alkyl sulfonamides comprise a series of recently synthesized compounds that have been identified as having antifungal properties [21,22,23] and antibacterial activity against *Erwinia carotovora Erwinia carotovora* subsp. *carotovora,* and *Xanthomonas axonopodis* pv. *Citri* [24]. A notable example of an alkyl sulfonamide is SYAUP-491, a 2-amido-cycloalkyl sulfonamide. The synthetic route for obtaining SYAUP-491 is shown in Figure 1. Using 4-n-propyl cyclohexanone (I) as the raw material, 2-oxy-4-n-propyl cyclohexyl sulfonate potassium salt (II) can be formed via a sulfonation reaction, and then turned into 2-oxy-4-n-propyl cyclohexyl sulfonamide compound (III) via oxalol chlorination and amination. This is then transformed into 2-amino-4-n-propyl cyclohexyl sulfonamide (IV) via reduction ammonification. Finally, the target compound SYAUP-491 is synthesized via condensation with trichloroacetyl chloride in the presence of triethylamine. The physical and chemical properties of SYAUP-491 are provided in Table 1. Details of compound SYAUP-491 can be found in the Supplementary Materials (Figures S1 and S2). In the present study, we comprehensively evaluated the antibacterial activity of SYAUP-491 against Xoo in vitro and in vivo. The mechanism underlying any antibacterial activity was then investigated using a series of experiments.

## 2. Results and Discussion

### 2.1. Antibacterial Activity In Vitro

As depicted in Table 2, the compound SYAUP-491 showed excellent activity against Xoo with an inhibition rate of 98.5% at a concentration of 100 μg/mL. Moreover, SYAUP-491 displayed stronger antibacterial activity than the commercially used control agents sulfadiazine, sulfisoxazole, thiodiazole copper, zhongshengmycin, zinc thiazole, and Cu(OH)_2_. This superior action was observed at all of the tested concentrations. To confirm the antibacterial activity of SYAUP-491, its EC_50_ value against Xoo was evaluated and found to be 6.96 μg/mL. This is higher than that of zinc thiazole and Cu(OH)_2_ (zinc thiazole: EC_50_ = 87.43 μg/mL and Cu(OH)_2_: EC_50_ = 102.92 μg/mL). In these experiments, streptomycin sulfate displayed the highest inhibition rate and lowest EC_50_ value.

At 50 μg/mL, SYAUP-491 resulted in a clear zone of inhibition against Xoo. Specifically, the inhibition zone diameter reached 16.8 mm, which was notably larger than that of Cu(OH)_2_ (average diameter of inhibition zone = 9.9 mm) and zinc thiazole (average diameter of inhibition zone = 7.6 mm) but smaller than that of streptomycin sulfate (average diameter of inhibition zone = 19.9 mm). These results are presented in Table 3.

### 2.2. Antibacterial Activity In Vivo

**Figure 2:**
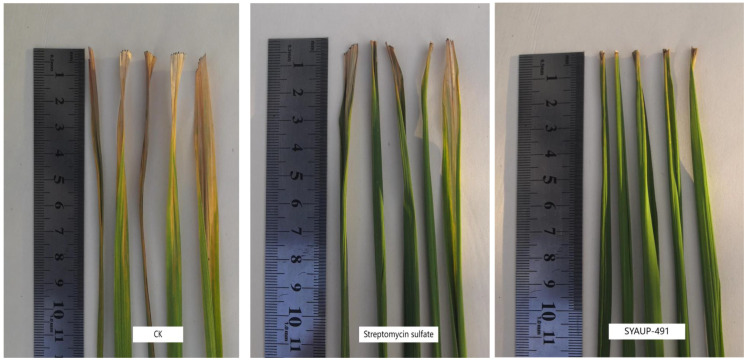
Curative activity of compound SYAUP-491 against *Xanthomonas oryzae* pv. *oryzae*.

All rice plants developed disease 7 days after leaf cutting, indicating the efficacy of the bacterial strain used in the experiment. As shown in Table 4 and Figure 2, the results show that the compound SYAUP-491 has both protective and curative effects in rice, and its curative effect is stronger than its protective effect. Moreover, SYAUP-491 showed excellent in vivo protective and curative effects against rice bacterial leaf blight (Xoo), with control effects of 32.2% and 74.1%, respectively, at 200 μg/mL, which were better than those of zinc thiazole (30.4% and 71.5%, respectively). Further, SYAUP-491 showed stronger curative effects than those of the control agent streptomycin sulfate.

Zinc thiazole is a novel fungicide that has a high efficiency and low toxicity [25]. In this study, the control agent zinc thiazole showed a low inhibitory rate in in vitro activity screening. The inhibitory rate of zinc thiazole at a 100 µg/mL concentration was only 57.2%, while in the live pot test, zinc thiazole showed good curative activity, with a curative effect of 71.5%. The results indicated that the activity of the compound in vitro and in vivo was not completely identical. Specifically, the toxicity of zinc thiazole against Xoo was not high in vitro, while strong inhibitory activity against Xoo was observed in vivo. This observation is consistent with the findings of previous studies [26].

Streptomycin is one of several antibiotics which have been used since the 1950s to control certain bacterial diseases, especially bacterial infections of high-value fruit, vegetables, and ornamental plants [27]. In this experiment, streptomycin sulfate was shown to work effectively in vitro. According to the findings, streptomyces 16S rRNA genes and the streptomycin resistance gene strA were not detected in agricultural streptomycin formulations extracted using an improved approach [28]. Producer organism resistance genes do not exist in agricultural streptomycin formulations. To date, no direct evidence has been found that resistance genes will be transferred to the bacteria itself. However, the rapid increase in antibiotic resistance, as well as efforts to preserve antibiotic efficacy in human medicine, has caused attention to be focused on nonmedical applications of antibiotics [29]. Concerns about antibiotic resistance in the environment have also led to bans or restrictions on the use of streptomycin in agriculture. Agricultural streptomycin products were officially withdrawn from the Chinese agricultural market on 15 June 2018. Therefore, there is an urgent need to develop new bactericidal compounds with high bacteriostatic activity. In this study, streptomycin sulfate showed strong protective and curative effects against Xoo in vitro. These effects were not as clear in vivo where it showed some protective activity, but relatively little curative activity. This discrepancy may be related to the frequency of application and the resistance of Xoo to streptomycin sulfate.

### 2.3. Membrane Permeability

**Figure 3:**
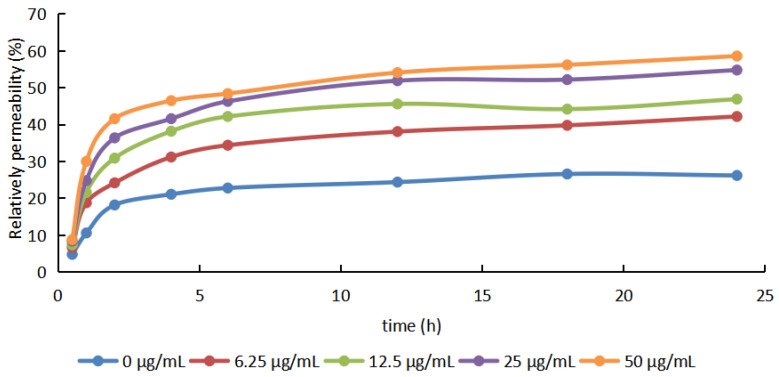
Effects of compound SYAUP-491 on cell membrane permeability.

As shown in Figure 3, the relative permeability of the Xoo cell membrane increased with increasing concentrations of SYAUP-491 and the time after treatment. The relative permeability in the presence of SYAUP-491 was also higher than that in the presence of the negative control (0 µg/mL). After treatment with SYAUP-491, the relative permeability of the cell membrane increased rapidly from 1 to 6 h and then increased slowly from 6 to 24 h, suggesting that SYAUP-491 damages the normal growth of Xoo bacterial cells by altering the permeability of the cell membrane.

### 2.4. Morphological Changes

**Figure 4:**
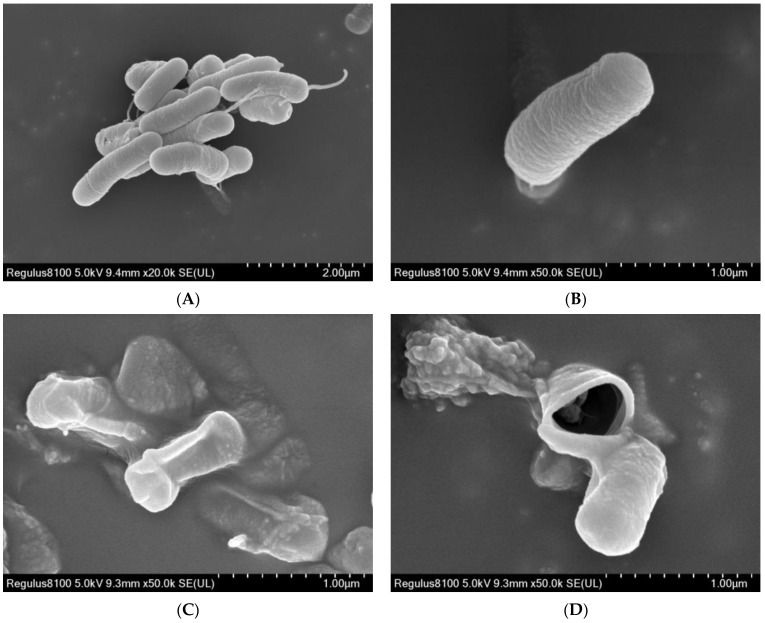
(**A**,**B**) Xoo based on SEM images after treatment with 0 μg/mL of SYAUP-491. (**C**,**D**) Xoo based on SEM images after treatment with 50 μg/mL of SYAUP-491.

Changes in the surface morphology and structure of the bacteria were observed via scanning electron microscopy(SEM) and transmission electron microscopy(TEM), respectively. Compared with observations of the negative control, treatment with 50 μg/mL of SYAUP-491 caused the bacterial bodies of Xoo to change from a uniform and full shape (Figure 4A,B) to being non-uniform, distorted, partially folded or ruptured, and with leakage holes on the surface of bacterial bodies (Figure 4C,D). The results showed that SYAUP-491 had a strong destructive effect on Xoo bacteria, eventually leading to their death.

**Figure 5:**
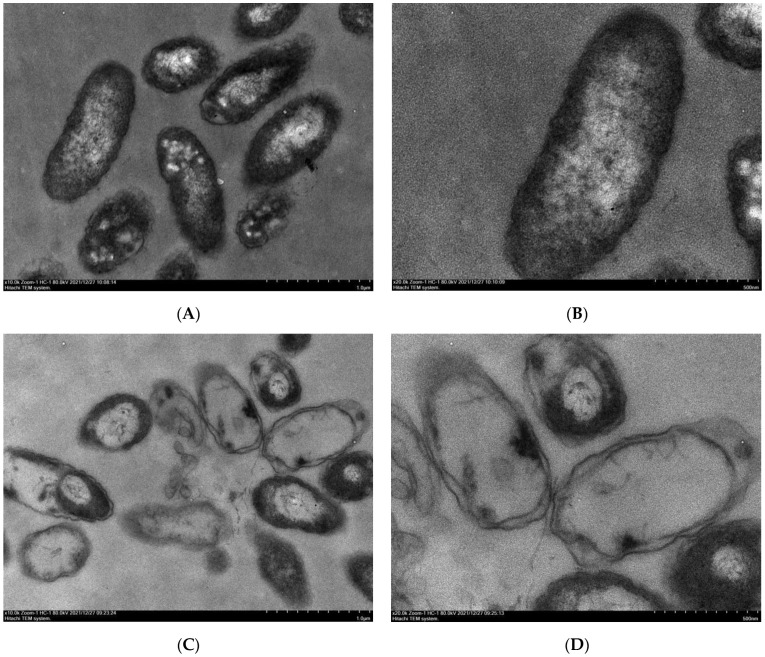
(**A**,**B**) Xoo based on TEM images after treatment with 0 μg/mL of SYAUP-491. (**C**,**D**) Xoo based on TEM images after treatment with 50 μg/mL of SYAUP-491.

The bacterial cells in the blank control group were complete, and the internal cytoplasm distribution was relatively uniform (Figure 5A,B). However, after treatment with SYAUP-491, the cells became deformed and shriveled, the internal cytoplasm was degraded and concentrated, and large vacuoles appeared (Figure 5C,D). Moreover, SYAUP-491 altered the permeability of the bacterial surface and the internal osmotic pressure within cells, thus inhibiting normal growth.

### 2.5. Analysis of Proteomic Results

#### 2.5.1. Global Analysis

As shown in Table 5, 560,926 secondary spectra were obtained using mass spectrometry. After searching the database, the number of available effective spectra was 307,012, and the utilization rate of the spectra was 54.7%. In total, 30,951 peptides and 2252 proteins were identified.

**Figure 6:**
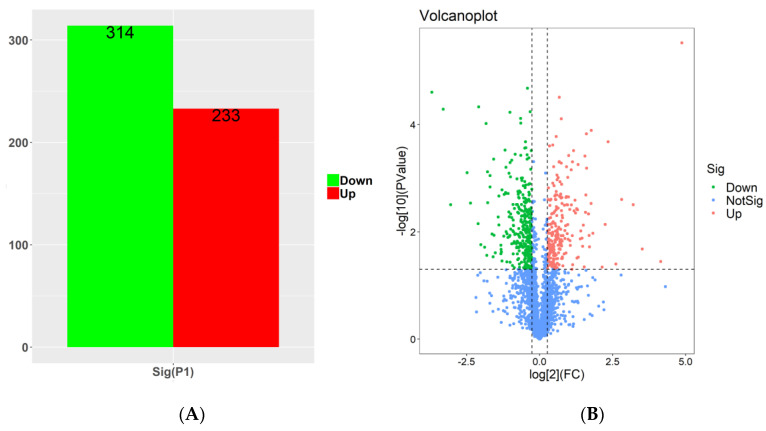
(**A**) Statistical analysis of the number of differentially expressed proteins in the samples. (**B**) Statistical analysis of the volcanoplot of different proteins in the samples. Note: The vertical dashed line represents *p* 0.05, while the horizontal dashed line represents FC 1.5 card value.

Differentially expressed proteins between the SYAUP-491 and CK groups were detected using t-statistics. Based on *p* < 0.05 (as well as an FDR value < 0.05) and an absolute fold-change > 1.2, we identified 233 proteins for which expression was upregulated and 314 for which expression was downregulated (Figure 6A,B). The red dots in the upper right corner represented upregulated differential proteins (FC ≥ 1.5), and the green dots in the upper left corner represented downregulated proteins (FC ≤ −1.5). In other words, the red dots represented upregulated proteins after the interaction of compound SYAUP-491 with bacteria, and their expression levels increased by at least 1.5 times, and the changes were statistically significant. The green dots represented proteins downregulated after treatment with the compound SYAUP-491, whose expression is reduced by at least 1.5 times, and the change was also statistically significant. The volcanoplot in Figure 6B allowed us to visually identify protein expression changes before and after treatment with the compound SYAUP-491, revealing those proteins that may interact with the compound and play an important role.

#### 2.5.2. Clusters of Orthologous Groups (COG) Functions

**Figure 7:**
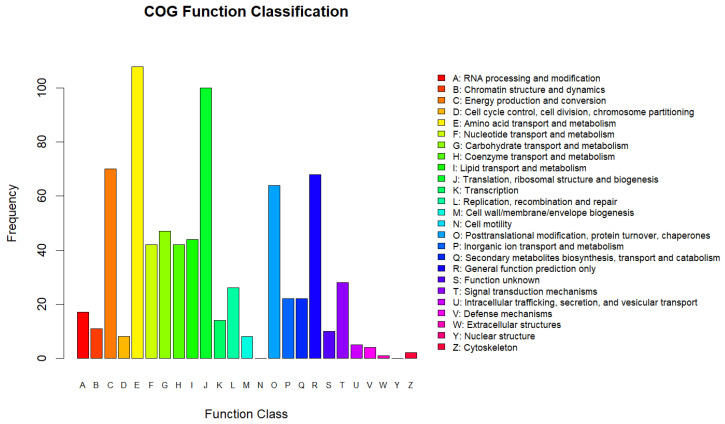
Statistical analysis of Clusters of Orthologous Groups (COG) functional classification.

We also searched the COG database to predict and classify the functions of the identified proteins. Overall, 2252 proteins were grouped into 25 COG functional classifications (Figure 7). The top five were amino acid transport and metabolism (E:108); translation, ribosomal structure, and biogenesis (J:100); energy production and conversion (C:70); general function prediction only (R:68); and post-translational modification, protein turnover, and chaperones (O:64).

#### 2.5.3. Gene Ontology (GO) Analysis

**Figure 8:**
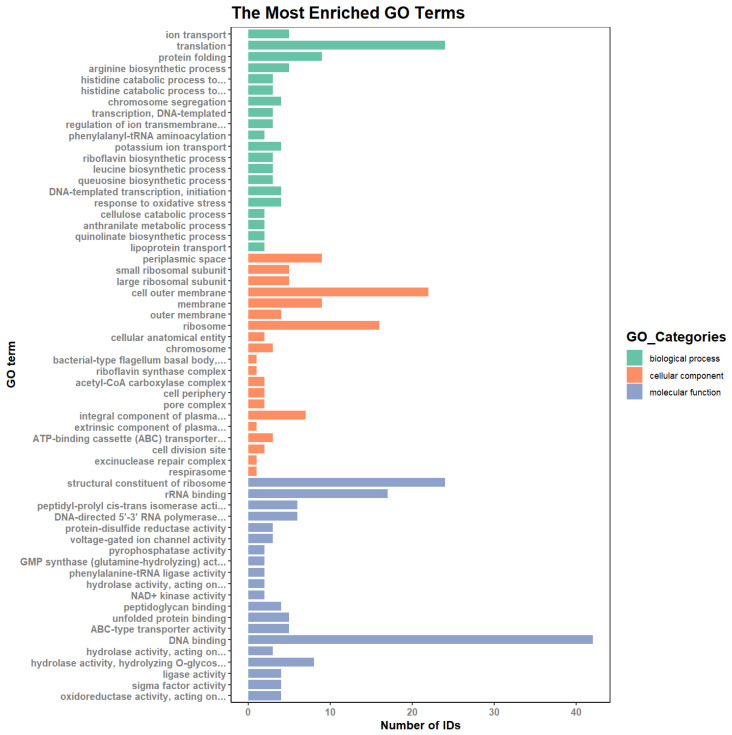
Gene Ontology (GO) analysis and classification of proteins.

We also conducted GO term enrichment analysis to group proteins with similar functions and associations. The differentially expressed proteins were enriched in several GO terms related to cellular components (CCs), biological processes (BPs), and molecular functions (MFs). The top 20 terms for these classifications were identified (Figure 8). Of these GO terms, translation, outer cell membrane, ribosome, DNA binding, and structural constituents of ribosomes were the five most enriched.

**Figure 9:**
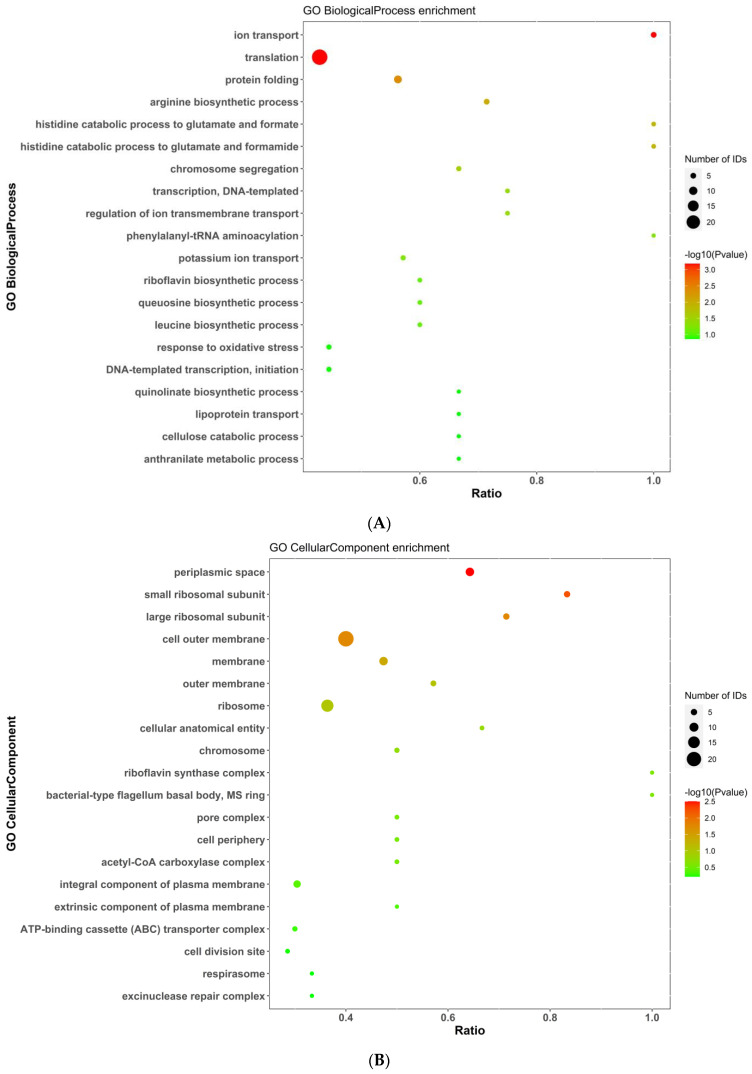

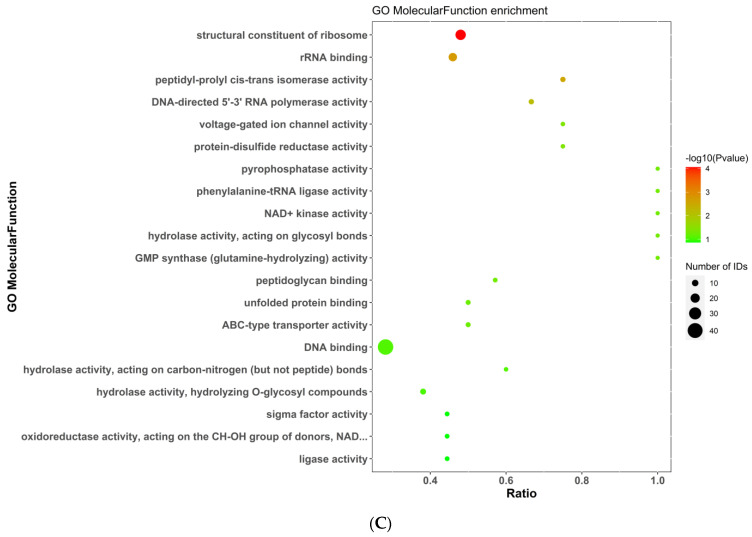
(**A**) Biological process (BP) enrichment analysis. (**B**) Cellular component (CC) enrichment analysis. (**C**) Molecular function (MF) enrichment analysis. Note: The figure shows the enrichment results for all identified proteins in terms of BPs, CCs, and MFs. Note: The abscissa is the ratio, the ordinate is each GO entry, the color represents the degree of enrichment (−log10(*p*-value)), and the circle size represents the number of genes.

The enrichment analysis of the differentially expressed proteins in terms of BPs, CCs, and MFs is represented by bubble diagrams in Figure 9A–C. In terms of BPs, the differentially expressed proteins were mainly enriched in translation and ion transport. In CCs, the differential proteins were mainly enriched in different terms, with the top three being periplasmic space, small ribosomal subunit, and cell outer membrane. Based on MF enrichment analysis, we concluded that the differentially expressed proteins were highly enriched in terms including structural constituents of ribosomes and rRNA binding, and the number of proteins enriched in the DNA-binding term was also relatively large.

#### 2.5.4. KEGG Analysis

**Figure 10:**
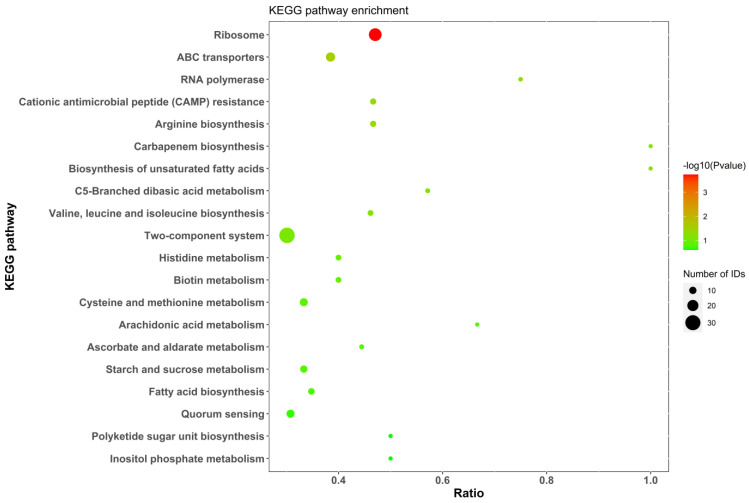
KEGG pathway enrichment analysis.

**Figure 11:**
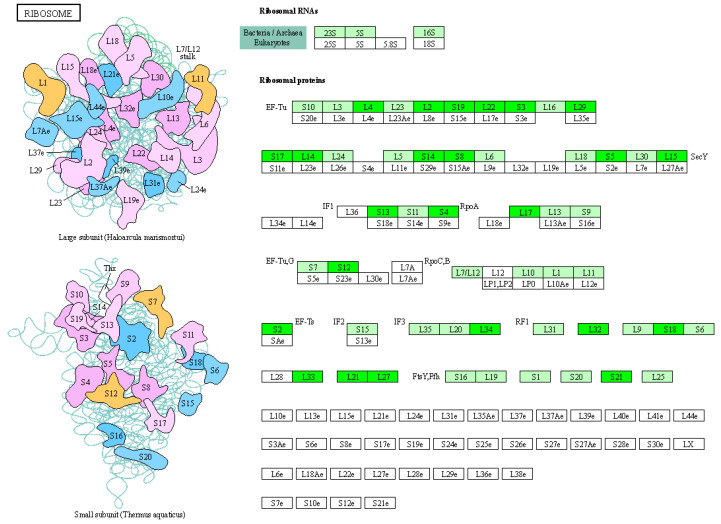
Ribosome pathway analysis. Note: The dark green color indicates that the expression of the protein was downregulated.

Figure 10 shows the enrichment results for all differentially expressed proteins in terms of KEGG pathways. The presence of a bubble represents a statistically significant result, the color of the bubble indicates the size of the *p*-value, and the size of the bubble represents the number of genes. The *X*-axis is the enrichment factor representing the ratio of differentially expressed proteins to total proteins in the measured pathways. Using the KEGG pathway annotations file and KEGG website (https://www.kegg.jp/kegg/pathway.html, accessed on 18 March 2024), we obtained the relevant pathway and map results of all differentially expressed proteins. The pathway with the highest enrichment of differential proteins was path:xoo03010; a schematic diagram of the ribosome results is shown in Figure 11.

The details of the specific differentially expressed proteins, based on the original data and the KEGG website, are shown in Table 6. The levels of all differentially expressed proteins were significantly downregulated, with smaller FC(SY_CK) values indicating a more significant downregulation. As shown in Table 6, the differentially expressed proteins were mainly enriched in two Xoo proteins, 50S ribosomal protein L33 and 50S ribosomal protein L34.

Proteomic analysis revealed a possible mechanism by which SYAUP-491 affects Xoo. These results suggest that SYAUP-491 may inhibit bacterial protein synthesis by disrupting the integrity of the bacterial cell membrane, altering the permeability of the bacterial cell membrane, acting on the 50S ribosomal subunit, and blocking the translation and ion transport process. However, further study is required to confirm the specific mechanisms involved.

The bactericidal mechanism of sulfonamide compounds is mainly via the inhibition of dihydrofolate synthetase in bacteria, thereby preventing bacteria from synthesizing dihydrofolate, a necessary substance for DNA and RNA, and eventually leading to bacterial death [30]. Although SYAUP-491 belongs to the alkyl sulfonamides, our proteomic results did not reveal significant effects on bacterial nucleic acid metabolic pathways. Some antibiotics, such as aminoglycosides, macrolides, tetracycline, and chloramphenicol, can bind to the 30S or 50S subunits of bacterial ribosomes, preventing peptide chain formation complex initiation and interfering with the binding of new amino acids to new peptide chains [31]. In summary, SYAUP-491 suppresses or kills bacteria by acting on a specific step in the protein-production process. The compound SYAUP-491 is likely to have a similar mechanism of action to the aforementioned antibiotics. According to the results of our proteomic analysis, the differential proteins of SYAUP-491 and CK were mainly enriched in the 30S and 50S subunits of Xoo ribosomes. Consequently, we hypothesize that SYAUP-491 may interact with or bind to certain proteins on the 50S or 30S subunits of Xoo ribosomes, thereby impeding translation and preventing the production of bacterial proteins. Further research is needed to test this hypothesis.

## 3. Materials and Methods

### 3.1. Materials and Instrument

Compound SYAUP-491 was synthesized by our laboratory. Streptomycin sulfate (original drug), sulfadiazine (original drug), and sulfisoxazole (original drug) were commercially available from “the molbase online mall” (Shanghai, China). Thiodiazole copper (20% suspension concentrates), zhongshengmycin (3% wettable powder), zinc thiazole (20% suspension concentrates), and Cu(OH)_2_ (46% water-dispersible granules) were purchased from local supermarkets (Shenyang, China). Enzyme label instrument (Spectra MAX 190, Meigu Molecular Instruments Co., Ltd., Shanghai, China) was used for testing of antibacterial activity in vitro. Scanning electron microscope (Regulus 8100, Hitachi Ltd., Tokyo, Japan) and transmission electron microscope (HT7700, Hitachi Ltd., Tokyo, Japan) were used for observing the morphological changes of the bacteria. Roteomic analysis was performed using the mass spectrometer (Orbitrap Exploris^TM^480, Thermo Fisher Technology Co., Ltd., Waltham, MA, USA).

### 3.2. Antibacterial Activity In Vitro

#### 3.2.1. Turbidimetry Test

The turbidimetry test was performed in accordance with a previously described protocol [32]. Dimethyl sulfoxide (DMSO) in sterile distilled water served as a blank control. Streptomycin sulfate (original drug), sulfadiazine (original drug), sulfisoxazole (original drug), thiodiazole copper (20% suspension concentrates), zhongshengmycin (3% wettable powder), zinc thiazole (20% suspension concentrates), and Cu(OH)_2_ (46% water-dispersible granules) were used as the positive controls. The synthesized target compound, SYAUP-491, was dissolved in DMSO and diluted with Luria–Bertani liquid medium in 96-well plates to obtain solutions of different concentrations (25 and 100 μg/mL). Experiments were performed in triplicate for each compound and control.

The 96-well plates were cultured at 28 °C for 48 h with shaking at 180 rpm. Following this, the optical density at 600 nm was measured, and the corrected OD values were obtained using an absorbance microplate reader. The inhibition rate was calculated using Equation (1), as follows:

Inhibition rate (%) = (corrected OD_CK_ − corrected OD_T_)/corrected OD_CK_ × 100 (1)

where corrected OD_CK_ is the corrected optical density of the blank control group and corrected OD_T_ is the optical density of the treated group. Based on previous antibacterial activity, the EC_50_ (median effective concentration) of some of the tested compounds against Xoo was evaluated using a doubling dilution. The experiments were performed in triplicate for each compound, and the results were processed using SPSS software (version 24.0).

#### 3.2.2. Inhibition Zone Test Using Filter Paper

We performed an inhibition zone test as described previously [33], although with a slight modification. Specifically, sterilized and dried filter paper with a diameter of 6 mm was placed onto the medium plate containing the bacterial liquid. Following this, 20 µL of the compound solution was absorbed using a microsyringe and added to the filter paper, such that the compound solution was diffused around the filter paper at the center. A blank control was added to each Petri dish. The inhibition zones were observed after 48 h. The target compound SYAUP-491 was dissolved in DMSO and diluted with water, and the effects were assessed at a final concentration of 50 µg/mL. DMSO in sterile distilled water was used as a blank control. Streptomycin sulfate, Cu(OH)_2_, and zinc thiazole were used as the positive controls. Each Petri dish contained two compound replicates and a blank control, and three Petri dish replicates were used for each test compound.

### 3.3. Antibacterial Activity In Vivo

The protective and curative activities of SYAUP-491 against Xoo were investigated in vivo using the leaf-cutting method [34] under greenhouse conditions. Streptomycin sulfate (original drug) and zinc thiazole (20% SC) were used as positive controls, and DMSO in sterile distilled water served as a negative control. Each treatment was performed in triplicate, and the concentrations of the active components of all positive controls were equivalent to those of SYAUP-491.

The rice variety tested was AD-10, which is susceptible to Xoo. In this experiment, the compounds (at concentrations of 200 µg/mL) were sprayed on rice leaves, and inoculation was performed after 24 h. The disease incidence was then assessed after 21 days. In the curative activity experiment, SYAUP-491, at a concentration of 200 µg/mL, was sprayed 7 days after inoculation. After 14 days, the disease incidence was checked and the length of the disease spot on the rice leaves was measured and recorded. The control effect was calculated using Equation (2) as follows:

C(%) = (L_CK_ − L_T_)/L_CK_ × 100% (2)

where C is the control effect, L_CK_ is the leaf lesion length of the negative control, and L_T_ is the leaf lesion length of the treated plants.

### 3.4. Membrane Permeability

Membrane permeability experiments were performed according to a previously reported method [35]. To remove the medium, 20 mL of the Xoo culture was centrifuged at 3000 rpm for 10 min. The cells were then collected, after which they were washed and resuspended twice in 20 mL of sterile water. Following this, the cells were treated with SYAUP-491 at concentrations of 0, 6.25, 12.5, 25, and 50 μg/mL. The same treatment with an equivalent volume of DMSO was used as the negative control. Conductivity was measured at 0, 0.5, 1, 2, 4, 6, 12, 18, and 24 h. Finally, conductivity was measured after the culture was boiled for 10 min to kill the Xoo cells. The relative permeability was calculated using Equation (3) as follows:

P = (C_t_ − C_0_)∕(C_k_ − C_0_) × 100% (3)

where P is the relative permeability, C_0_ is the conductivity at 0 min, C_t_ is the conductivity at different time points, and C_k_ is the conductivity after cell death.

### 3.5. Morphological Changes

Changes in cell morphology were observed using scanning electron microscopy (SEM) [36] and transmission electron microscopy (TEM). The Xoo, which were cultured to the logarithmic growth phase, were centrifuged (28 °C, 180 rpm) and twice washed with phosphate-buffered saline (0.1 M PBS, pH = 7.2–7.4). The cells were then treated with SYAUP-491 at concentrations of 0 and 50 μg/mL. After incubation at 28 °C for 12 h, the bacteria were further suspended in 0.1 M PBS buffer (pH 7.2–7.4) and then transferred into a 1.5 mL centrifuge tube for further centrifugation (3000 rpm, 10 min) to collect the bacteria. After a series of treatments including fixation, dehydration, lyophilization, and gold coating, the samples were visualized using SEM. The samples were then placed onto a transmission electron microscope for observation and photography after fixation, rinsing, gradient dehydration, embedding, immersion, polymerization, ultrathin sectioning, and staining.

### 3.6. Proteomic Analysis of the Effects of Compound SYAUP-491 on Xoo

To investigate the potential mechanism underlying the antibacterial effect of SYAUP-491 on Xoo, differentially expressed proteins were detected using label-free quantitative proteomic techniques. In this study, two experimental groups, SYAUP-491 (50 μg/mL) and CK, were established, and each group was tested three times to increase the reliability of the results. The samples were incubated with the compounds, and the total protein was extracted first, followed by trypsin digestion. LC-MS/MS was used for mass spectrometry. The database search was performed using Proteome Discoverer 2.4 software while data processing and analysis were conducted using the UNIPROT database.

## 4. Conclusions

SYAUP-491 is a novel alkyl sulfonamide that shows strong effects against Xoo both in vitro and in vivo. Broadly, the basis of this activity involves an alteration in the membrane permeability and subsequent morphological disruption. Further, an analysis of the proteomic results suggests that these effects are caused by the inhibition of bacterial protein synthesis mediated by disrupting the integrity of the bacterial cell membrane, altering the permeability of the bacterial cell membrane, and acting on ribosomes in the bacterial body. In conclusion, SYAUP-491 can be considered a new lead compound in antibacterial research and its capabilities as a commercial drug should be further investigated. However, further studies should be conducted at the molecular level to reveal the detailed mechanism via which SYAUP-491 inhibits Xoo.

## Figures and Tables

**Table 1:** Physical and chemical properties of the compound SYAUP-491.

Molecular Weight and Formula	State	Melting Point (°C)	Yield (%)	^1^H NMR (CDCl_3_, δ)	IR (ν, cm^−1^)	HRMS (z/e)
544.23 C_18_H_21_Cl_4_F_3_N_2_O_3_S	White crystal	123–125	98.5	0.85 (t, *J* = 7.1 Hz, 3H, CH_3_), 2.34–1.18 (m, 11H, C_6_H_12_), 3.83 (t, *J* = 10.3 Hz, 1H, CH-N), 4.17 (td, *J* = 7.1, 3.7 Hz, 1H, CH-SO_2_), 7.55–7.82 (m, 3H, Ph-H), 8.67 (d, *J* = 8.4 Hz, 1H, CO-NH), 9.80 (s, 1H, SO_2_-NH)	3435, 3336, 2958, 2873, 1689	543.0047 (M + H)

**Table 2:** In vitro antibacterial activities of the compounds tested against Xanthomonas *oryzae* pv. *oryzae*.

Compound	Inhibition Rate (%)		Regression Equation	EC_50_ (μg/mL)
	100 (μg/mL)	25 (μg/mL)		
SYAUP-491	98.5 ± 1.2 a	81.2 ± 1.6 b	y = 1.3193x + 3.8884	6.96
Streptomycin sulfate	100 a	95.2 ± 3.0 a	y = 1.9398x + 3.8441	3.94
Sulfadiazine	36.6 ± 4.0 d	18.9 ± 5.7 d	—	—
Sulfisoxazole	38.1 ± 2.7 d	22.3 ± 2.9 d	—	—
Thiodiazole copper	29.7 ± 4.9 e	11.7 ± 3.2 e	—	—
Zhongshengmycin	19.6 ± 5.6 f	6.9 ± 4.2 e	—	—
Zinc thiazole	57.2 ± 4.1 b	35.0 ± 2.7 c	y = 0.5639x + 3.9052	87.43
Cu(OH)_2_	47.9 ± 4.7 c	36.2 ± 4.5 c	y = 0.4967x + 3.9014	102.92

Note: The experiments were repeated three times. Values are means ± SDs. ‘—’ means no data. In each subcolumn, data marked with different lowercase letters differ significantly at *p* ≤ 0.05 according to the post hoc pairwise comparisons.

**Table 3:** Inhibition zone diameters of tested compounds against *Xanthomonas oryzae* pv. *oryzae* at 50 μg/mL based on the filter paper method.

Compound	Mean IZD (mm)
SYAUP-491	16.8 ± 1.1 b
Streptomycin sulfate	19.9 ± 0.3 a
Cu(OH)_2_	9.9 ± 0.5 c
Zinc thiazole	7.6 ± 0.6 d
CK	—

Note: “IZD” means inhibition zone diameter. “—” in the table indicates that no inhibition zone is generated. In each subcolumn, data marked with different lowercase letters differ significantly at *p* ≤ 0.05 according to the post hoc pairwise comparisons.

**Table 4:** Preventive and curative activity of compound SYAUP-491.

Compound	Morbidity (%)	Protective Activity (%)	Curative Activity (%)
SYAUP-491	100	32.2 ± 3.3 b	74.1 ± 6.3 a
Streptomycin sulfate	100	50.1 ± 3.4 a	38.8 ± 2.8 b
Zinc thiazole	100	30.4 ± 6.6 b	71.5 ± 7.5 a
CK	100	/	/

Note: In each subcolumn, data marked with different lowercase letters differ significantly at *p* ≤ 0.05 according to the post hoc pairwise comparisons.

**Table 5:** Statistics of protein-identification results.

Name	Total Spectra	Matched Spectrum	Peptide	Identified Protein
Total	560,926	307,012	30,951	2252

**Table 6:** Specific information of differential expression proteins in the ribosomal pathway.

ID	Accession	Gene Name	Description	KO	FC(SY_CK)	*p*-Value (SY_CK)
RS3_XANOR	Q5GWU0	*rpsC*	30S ribosomal protein S3	xoo:XOO3577	0.742866	0.047873
RL21_XANOR	Q5H2E7	*rplU*	50S ribosomal protein L21	xoo:XOO1620	0.8138	0.047358
RS18_XANOR	Q5H052	*rpsR*	30S ribosomal protein S18	xoo:XOO2415	0.624458	0.012651
RS5_XANOR	Q5GWV1	*rpsE*	30S ribosomal protein S5	xoo:XOO3566	0.820401	0.021934
RL15_XANOR	Q5GWV4	*rplO*	50S ribosomal protein L15	xoo:XOO3563	0.677525	0.009118
RL17_XANOR	Q5GWW0	*rplQ*	50S ribosomal protein L17	xoo:XOO3557	0.733943	0.002582
RL14_XANOR	Q5GWU4	*rplN*	50S ribosomal protein L14	xoo:XOO3573	0.789138	0.001995
RS17_XANOR	Q5GWU3	*rpsQ*	30S ribosomal protein S17	xoo:XOO3574	0.781231	0.032046
RS21_XANOR	Q05I90	*rpsU*	30S ribosomal protein S21	xoo:XOO4929	0.607631	0.031546
RL4_XANOR	Q5GWT5	*rplD*	50S ribosomal protein L4	xoo:XOO3582	0.7409	0.0078
RS8_XANOR	Q05HS7	*rpsH*	30S ribosomal protein S8	xoo:XOO4896	0.692477	0.002344
RS4_XANOR	Q5GWV8	*rpsD*	30S ribosomal protein S4	xoo:XOO3559	0.720856	0.00134
RS14_XANOR	Q5GWU8	*rpsN*	30S ribosomal protein S14	xoo:XOO3569	0.711797	0.031258
RS12_XANOR	Q5GWS8	*rpsL*	30S ribosomal protein S12	xoo:XOO3589	0.493916	0.01166
RS13_XANOR	Q5GWV6	*rpsM*	30S ribosomal protein S13	xoo:XOO3561	0.670199	0.007928
RS2_XANOR	Q5H1E0	*rpsB*	30S ribosomal protein S2	xoo:XOO1977	0.649435	0.001001
RL27_XANOR	Q5H2E6	*rpmA*	50S ribosomal protein L27	xoo:XOO1621	0.639016	0.000094
RL22_XANOR	Q5GWT9	*rplV*	50S ribosomal protein L22	xoo:XOO3578	0.701991	0.00116
RS19_XANOR	Q5GWT8	*rpsS*	30S ribosomal protein S19	xoo:XOO3579	0.731434	0.014835
RL2_XANOR	Q5GWT7	*rplB*	50S ribosomal protein L2	xoo:XOO3580	0.635694	0.018403
RL32_XANOR	Q05I49	*rpmF*	50S ribosomal protein L32	xoo:XOO4732	0.701572	0.036503
RL29_XANOR	Q5GWU2	*rpmC*	50S ribosomal protein L29	xoo:XOO3575	0.446445	0.001933
RL34_XANOR	Q05HP6	*rpmH*	50S ribosomal protein L34	xoo:XOO4957	0.121112	0.003118
RL33_XANOR	Q5GU11	*rpmG*	50S ribosomal protein L33	xoo:XOO4558	0.101754	0.000052

## Data Availability

Data will be made available upon request to the corresponding author.

## Supplementary Materials

The following supporting information can be downloaded at: https://www.mdpi.com/article/10.3390/molecules29061413/s1. Figure S1: 1H NMR spectrum of compound SYAUP-491. Figure S2: HRMS spectrum of compound SYAUP-491.
