# Supplementary material for: Antibacterial Activities and Underlying Mechanisms of the Compound SYAUP-491 against Xanthomonas oryzae pv. oryzae
Source: Molecules. 2024 Mar 21;29(6):1413. doi: 10.3390/molecules29061413 (PMC10974988; doi:10.3390/molecules29061413)
Supplement: Supplementary file 1 [file molecules-29-01413-s001.zip › Figure S1. 1H NMR spectrum of compound SYAUP-491.pdf]

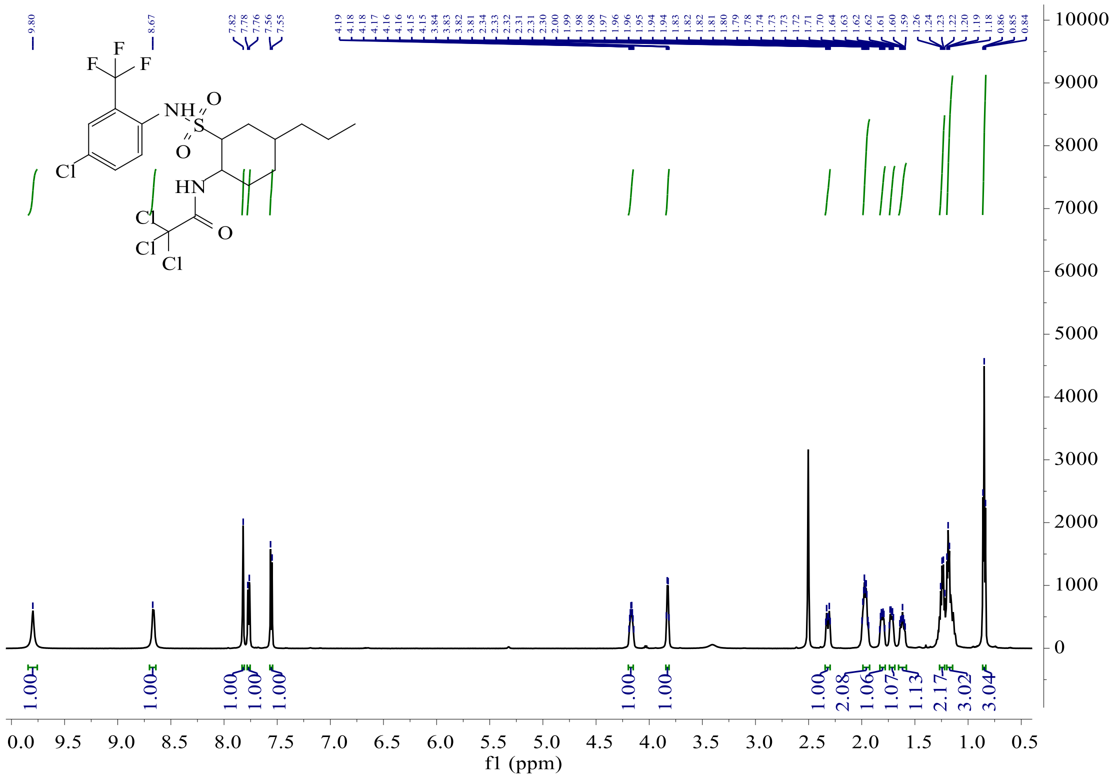

Figure S1. ^{1}H NMR spectrum of compound SYAUP-491

^{1}H NMR (600 MHz, DMSO-d_6) δ 0.85 (t, J = 7.1 Hz, 3H, CH_3), 2.34-1.18 (m, 11H, C_6H_{12}), 3.83 (t, *J* = 10.3 Hz, 1H, CH-N), 4.17 (td, *J* = 7.1, 3.7 Hz, 1H, CH-SO_2), 7.55–7.82 (m, 3H, Ph-H), 8.67 (d, J = 8.4 Hz, 1H, CO-NH), 9.80 (s, 1H, SO_2-NH)
