# Supplementary material for: Antibacterial Activities and Underlying Mechanisms of the Compound SYAUP-491 against Xanthomonas oryzae pv. oryzae
Source: Molecules. 2024 Mar 21;29(6):1413. doi: 10.3390/molecules29061413 (PMC10974988; doi:10.3390/molecules29061413)
Supplement: Supplementary file 1 [file molecules-29-01413-s001.zip › Figure S2. HRMS spectrum of compound SYAUP-491.pdf]

Product	Molecular weight	Molecular Formula	Precise molecular weight	M+H	Measurement value	Deviation ppm
SYAUP-491	544.23	$C_{18}H_{21}Cl_4F_3N_2O_3S$	541.9979	543.0052	543.0047	-0.9

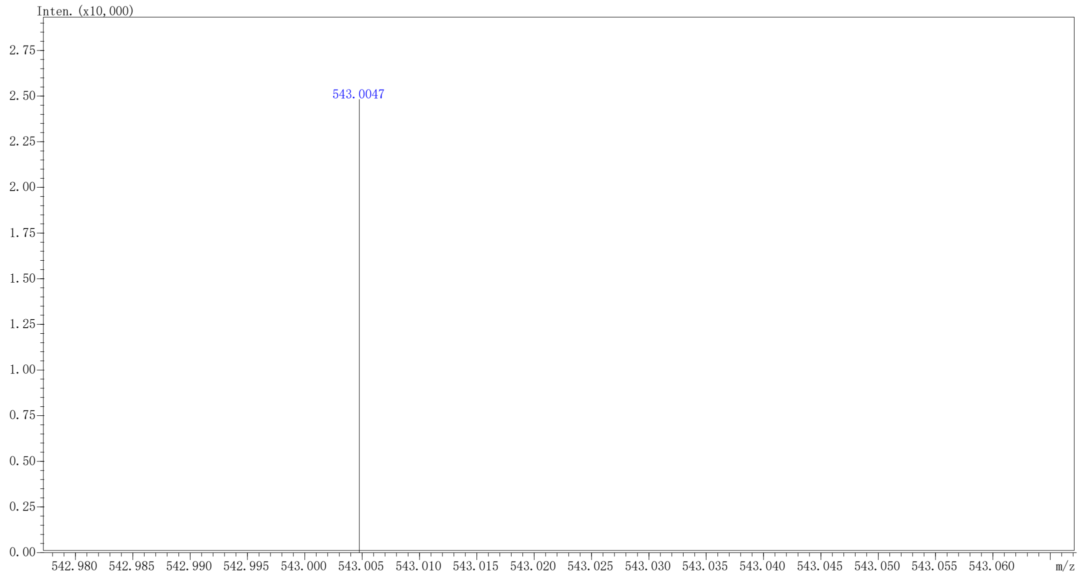

Figure S2. HRMS spectrum of compound SYAUP-491
